# Structural Health Monitoring of Defective Carbon Fiber Reinforced Polymer Composites Based on Multi-Sensor Technology

**DOI:** 10.3390/s25175259

**Published:** 2025-08-24

**Authors:** Wuyi Li, Heng Huang, Boli Wan, Xiwen Pang, Guang Yan

**Affiliations:** 1 College of Aerospace Engineering, Nanjing University of Aeronautics and Astronautics, Nanjing 210016, China; liwuyi1982@163.com; 2 College of Instrument Science and Opto-Electronics Engineering, Beijing Information Science & Technology University, Beijing 100101, Chinapangxw4977@163.com (X.P.)

**Keywords:** carbon fiber reinforced polymer, fiber Bragg grating, electrical resistance strain gauge, damage monitoring, finite element simulation

## Abstract

Carbon fiber reinforced polymer (CFRP) composites are prone to developing localized material loss defects during long-term service, which can severely degrade their mechanical properties and structural reliability. To address this issue, this study proposes a multi-sensor synchronous monitoring method combining embedded fiber Bragg grating (FBG) sensors and surface-mounted electrical resistance strain gauges. First, finite element simulations based on the three-dimensional Hashin damage criterion were performed to simulate the damage initiation and propagation processes in CFRP laminates, revealing the complete damage evolution mechanism from initial defect formation to progressive failure. The simulations were also used to determine the optimal sensor placement strategy. Subsequently, tensile test specimens with prefabricated defects were prepared in accordance with ASTM D3039, and multi-sensor monitoring techniques were employed to capture multi-parameter, dynamic data throughout the damage evolution process. The experimental results indicate that embedded FBG sensors and surface-mounted strain gauges can effectively monitor localized material loss defects within composite laminate structures. Strain gauge measurements showed uniform strain distribution at all measuring points in intact specimens (with deviations less than 5%). In contrast, in defective specimens, strain values at measurement points near the notch edge were significantly higher than those in regions farther from the notch, indicating that the prefabricated defect disrupted fiber continuity and induced stress redistribution. The combined use of surface-mounted strain gauges and embedded FBG sensors was demonstrated to accurately and reliably track the damage evolution behavior of defective CFRP laminates.

## 1. Introduction

Carbon fiber reinforced polymer composites have gradually become key materials in the manufacturing of primary load-bearing components for aerospace vehicles due to their outstanding specific strength and stiffness, excellent structural designability, superior wear resistance, and remarkable corrosion resistance [1,2,3,4,5,6]. However, during long-term service, external factors such as bird strikes, tool impacts, sustained external loads, and complex operational environments inevitably lead to localized material loss defects in composite structures. These defects cause significant degradation of the load-bearing capacity of aerospace vehicles, posing serious threats to flight safety. Moreover, as service time increases, damage in the material loss regions accumulates and propagates, inducing various damage modes such as matrix cracking, fiber fracture, and interlaminar delamination, which further compromise the overall structural performance and reduce service reliability. Therefore, accurate monitoring of localized material loss defects in CFRP laminates and in-depth investigation of the damage evolution laws under defect conditions are of great engineering significance and practical value for improving flight safety, controlling ultimate speed, and extending the service life of aerospace composite structures.

Conventional methods for monitoring structural damage in composite laminates include ultrasonic C-scanning [7], X-ray inspection [8], thermography [9], fiber Bragg gratings [10], and strain gauges [11]. These techniques enable damage detection while maintaining the structural integrity of composite components and are thus widely employed in the industry. Among these, ultrasonic inspection has long been the industry standard for structural health assessment of fiber-reinforced polymers (FRPs), owing to its short inspection time and strong defect detection capabilities [12]. However, the deployment of ultrasonic systems is relatively complex and not suitable for real-time, continuous monitoring of composite structures. In contrast, techniques such as FBG sensors and strain gauges offer promising potential for online, real-time monitoring of the structural health state of composite materials, making them increasingly attractive alternatives to conventional ultrasonic C-scanning methods [13,14].

Fiber Bragg grating sensors offer numerous technical advantages, including excellent immunity to electromagnetic interference, small size enabling easy embedding within composite materials, long service life, and high safety levels [15], making them widely applicable in composite structural damage monitoring. Schizas et al. [16] developed an FBG-based multiplexed sensing system capable of effectively detecting debonding and delamination failures in composite structures. Rong et al. [17] proposed a method for detecting composite delamination using surface-mounted FBG sensors by measuring static strain to accurately identify delaminated regions. Goossens et al. [18] employed a network of 120 FBG sensors to monitor vulnerable stiffener feet on large CFRP panels and successfully detected and localized barely visible impact damage (BVID), demonstrating the potential of fiber-optic sensor networks for aerospace composite damage monitoring. Wang et al. [19] embedded FBG sensors into CFRP laminates to monitor strain in wind turbine blades. Rocha et al. [20] compared the detection performance of FBG sensors embedded at different depths within CFRP for barely visible impact damage, showing that FBG sensors could capture both residual strains after damage and strain peaks during impact events. Prusty and Raju [21] proposed a damage detection method for offshore composite structures using a combination of acoustic emission (AE) and FBG sensors. Their results demonstrated that FBG sensors could accurately detect damage initiation, progression, magnitude, and location within composite structures through sudden strain fluctuations or sharp strain gradient spikes. Freire et al. [22] investigated the potential of FBG sensors as strain measurement devices in composite-reinforced industrial steel pipe applications. Li et al. [23] applied FBG sensors for structural health monitoring (SHM) of three-dimensional woven composite structures. Similarly, Li et al. [24] utilized FBG strain sensors for SHM of 3D six-directional braided composites. Wu et al. [25] integrated FBG sensors with piezoelectric transducers (PZTs) and demonstrated through experimental testing that the system could effectively monitor damage in composite plates.

In summary, existing research has demonstrated significant progress in monitoring the strain and damage of composite laminates using fiber Bragg grating sensors and electrical resistance strain gauges. However, most of these studies have focused on defect-free composite laminates [16,17,18], while investigations on damage monitoring for defective laminates remain relatively limited. To address this gap, this study focuses on CFRP laminates with prefabricated defects. Firstly, finite element simulations are conducted to preliminarily analyze the damage evolution behavior of defective CFRP laminates and to determine the optimal locations for embedded FBG sensors. Based on this, a multi-sensor synchronous monitoring approach is proposed, combining embedded FBG sensors, which are well suited for internal integration within composites, with surface-mounted strain gauges, which are cost-effective and easy to apply. Tensile tests are performed on defective CFRP laminates using this multi-sensor configuration, and the results are compared with those from intact laminates under identical loading conditions. The experimental results indicate that this method can effectively detect the presence of defects and reveal that the introduction of defects significantly alters the damage evolution behavior of the laminates. Furthermore, by comparing the experimental results with finite element predictions, the proposed multi-sensor synchronous monitoring method is validated to effectively capture the damage evolution processes at different locations both on the surface and inside defective laminates, providing valuable experimental evidence and methodological support for damage monitoring of defective composite structures in service.

## 2. Principle

### 2.1. Fiber Bragg Grating Sensor Principle

The fabrication of fiber Bragg gratings involves the precise, periodic exposure of the core of a single-mode optical fiber to intense ultraviolet (UV) light. This UV exposure induces a localized change in the refractive index within the fiber core through chemical bond rearrangement and modifications in the physical structure of the silica-based material, resulting in the formation of a periodic refractive index modulation—known as a grating. This grating structure selectively reflects light of specific wavelengths according to Bragg’s law, while allowing other wavelengths to transmit through the fiber. This property makes FBGs highly effective as both optical sensors and communication components.

When all reflected light waves undergo coherent superposition, a strong reflected signal with a specific wavelength characteristic—known as the Bragg condition—is formed. The incident wavelength that satisfies this condition is defined as the Bragg wavelength. Light signals at non-Bragg wavelengths experience minimal modulation from the fiber grating and are able to propagate through the fiber with little interference. The condition for satisfying the Bragg wavelength can be expressed by the following mathematical equation:

(1) $$\lambda_{B}={2n}_{\mathrm{eff}}\Lambda$$

where $\lambda_{B}$ represents the Bragg wavelength of the FBG, $n_{\mathrm{eff}}$ is the effective refractive index of the fiber core, and Λ is the grating period. When an FBG sensor is subjected to strain or temperature changes, the Bragg wavelength shifts accordingly [26], as expressed in Equation (2):

(2) $$\frac{\Delta \lambda_{B}}{\lambda_{B}}=(1-P_{e})\Delta \varepsilon +(\alpha +\xi )\Delta T$$

where $\Delta \lambda_{B}$ is the Bragg wavelength shift, Δε is the strain variation, $P_{e}$ is the effective photo-elastic coefficient, α is the thermal expansion coefficient, and ξ is the thermo-optic coefficient.

If the experiment is conducted under constant temperature conditions, or if temperature compensation is applied, the relationship between the Bragg wavelength shift and strain can be simplified as shown in Equation (3):

(3) $$\frac{\Delta \lambda_{B}}{\lambda_{B}}=(1-P_{e})\Delta \varepsilon$$

where $P_{e}$ is a constant. As indicated by Equation (3), under isothermal conditions, the Bragg wavelength shift is linearly proportional to the strain variation, enabling fiber Bragg gratings to function effectively as strain sensing elements.

### 2.2. Working Principle of Electrical Resistance Strain Gauges

Here, electrical resistance strain gauges are sensing elements that operate based on the change in electrical resistance resulting from mechanical deformation. Their structure typically consists of four basic components: a substrate, a sensitive grid, a protective covering layer, and lead wires. The metallic resistance strain effect describes how the resistance of a metallic material changes when it undergoes mechanical strain due to applied external forces. The strain gauge utilizes this physical phenomenon by converting the deformation of the metallic sensitive grid under mechanical load into a corresponding change in electrical resistance. Thus, by accurately measuring the change in resistance caused by strain, the corresponding strain value can be precisely calculated.

For a metallic wire with length L, cross-sectional area A, and electrical resistivity ρ, its resistance R can be calculated using Equation (4) [27]:

(4) $$R=\rho \frac{L}{A}$$

When the sensitive metal grid undergoes tension or compression, its resistivity ρ, length L, and cross-sectional area A all change with the deformation, resulting in a resistance variation ΔR. Through differential calculation, the relationship between relative resistance change and strain can be expressed by Equation (5):

(5) $$\frac{\Delta R}{R}=K_{0}\varepsilon$$

where $K_{0}$ represents the gauge factor of the metal sensitive grid, indicating the sensitivity of the strain gauge to strain. Its physical meaning is the ratio of the relative change in resistance to the mechanical strain when the sensitive grid undergoes a unit change in length.

**Figure 1:**
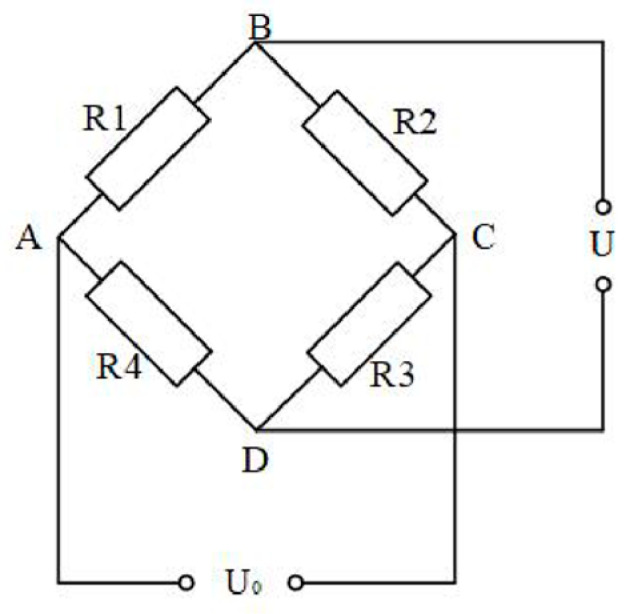
Schematic diagram of a Wheatstone bridge.

For precise measurement of resistance changes, the Wheatstone bridge circuit is typically employed. The Wheatstone bridge can convert changes in circuit resistance into output voltage variations. The Wheatstone bridge has quarter-bridge, half-bridge, and full-bridge configurations. The quarter-bridge is suitable for unidirectional strain measurement, the half-bridge offers higher sensitivity than the quarter-bridge and provides temperature self-compensation, while the full-bridge has the highest sensitivity and the strongest anti-interference capability. This study involves only unidirectional strain measurement and the experimental duration is very short with the specimen and room temperature remaining stable during this period. Therefore, the quarter-bridge configuration is selected for measurement. The schematic diagram of the Wheatstone bridge is shown in Figure 1.The working principle of the quarter-bridge is as follows: R1 is the resistance strain gauge, R2, R3, and R4 are the bridge arm resistors, U0 is the excitation voltage, and U is the output measured signal voltage.

When the resistance R_1_ of the strain gauge changes by an amount ΔR, the output voltage U of the Wheatstone bridge can be calculated using the following equation:

(6) $$U=\frac{(R1+\Delta R)\cdot R3-R2\cdot R4}{\left(R1+\Delta R+R2)\cdot (R3+R4\right)}\cdot U_{0}$$

Since the strain gauge detects very small deformations of the material, the change in resistance ΔR is much smaller than R_1_ (ΔR ≪ R_1_). Moreover, when all resistances in the bridge are equal (R_1_ = R_2_ = R_3_ = R_4_ = R), the equation for the output voltage U can be simplified to:

(7) $$U=\frac{1}{4}\cdot \frac{∆R}{R}\cdot U_{0}=\frac{1}{4}K_{0}\cdot \varepsilon \cdot U_{0}$$

where *K*_0_ is the gauge factor (strain sensitivity coefficient) of the strain gauge and ε is the measured strain.

### 2.3. Three-Dimensional Hashin Failure Criteria

In practical applications, composite materials are often subjected to complex three-dimensional loading conditions. Their damage modes involve not only fiber and matrix failures within individual plies but also interlaminar interface damage. Therefore, conventional failure criteria based solely on stress or strain are insufficient for comprehensively evaluating the damage evolution process, and traditional two-dimensional failure criteria struggle to account for interlaminar effects.

The three-dimensional Hashin failure criteria [28], by incorporating both the anisotropic characteristics of composite materials and multiple failure mechanisms, introduces the capability to describe interlaminar damage, enabling more accurate predictions of damage modes and failure processes in composites. Accordingly, this study adopts the 3D Hashin failure criteria as the damage evaluation standard to enhance the reliability of the simulation results and better reflect the actual damage evolution behavior of composite laminates. The failure expressions used in this paper are as follows:

1. Fiber tensile failure ($\sigma_{11}$ > 0)
  (8) $$F_{ft}={\left(\frac{\sigma_{11}}{X_{t}}\right)}^{2}+{\left(\frac{\tau_{12}}{S_{12}}\right)}^{2}+{\left(\frac{\tau_{13}}{S_{13}}\right)}^{2}\geq 1$$
2. Fiber compression failure ($\sigma_{11}$ < 0)
  (9) $$F_{fc}={\left(\frac{\sigma_{11}}{X_{c}}\right)}^{2}\geq 1$$
3. Matrix tensile failure ($\sigma_{22}+\sigma_{33}>0$)
  (10) $$F_{mt}={\left(\frac{\sigma_{22}+\sigma_{33}}{Y_{t}}\right)}^{2}+{\left(\frac{\tau_{12}}{S_{12}}\right)}^{2}+{\left(\frac{\tau_{13}}{S_{13}}\right)}^{2}+\left(\frac{1}{S_{23}^{2}}\right)(\tau_{23}^{2}-\sigma_{22}\sigma_{33})\geq 1$$
4. Matrix compression failure ($\sigma_{22}+\sigma_{33}<0$)
  (11) $$F_{mc}={\left(\frac{\sigma_{22}+\sigma_{33}}{{2S}_{23}}\right)}^{2}+\left(\frac{\sigma_{22}+\sigma_{33}}{Y_{c}}\right)\left[{\left(\frac{Y_{c}}{2S_{23}}\right)}^{2}-1\right]+\frac{1}{S_{23}^{2}}\left(\tau_{23}^{2}-\sigma_{22}\sigma_{33}\right)+{\left(\frac{\tau_{12}}{S_{12}}\right)}^{2}+{\left(\frac{\tau_{13}}{S_{13}}\right)}^{2}\geq 1$$

**Figure 2:**
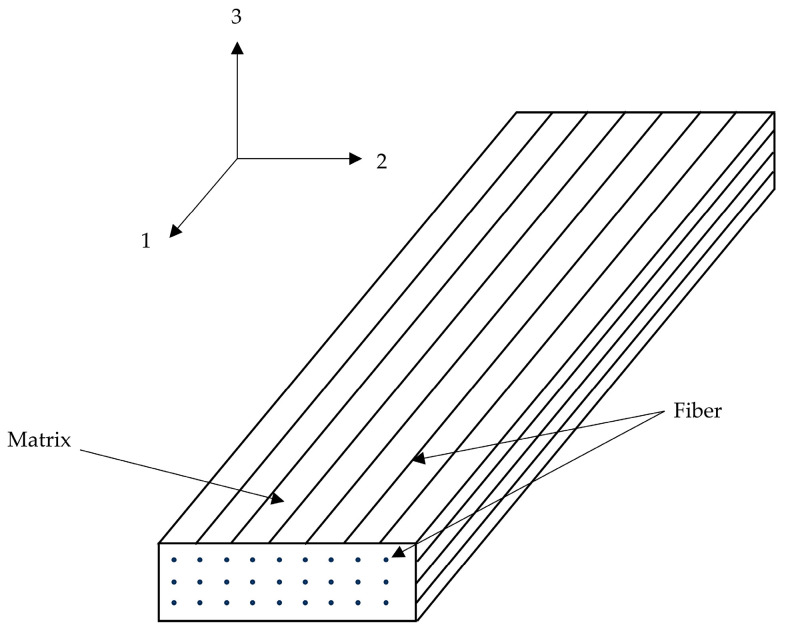
Schematic diagram of CFRP structure.

In the aforementioned given expressions, the indices 1, 2, and 3 correspond to the principal material directions: 1 represents the fiber orientation, 2 denotes the in-plane transverse direction, and 3 indicates the out-of-plane transverse direction, as shown in Figure 2. The normal stress components are defined as $\sigma_{11}$, $\sigma_{22}$, and $\sigma_{33}$ (positive values indicate tension), while $\tau_{12}$, $\tau_{13}$, and $\tau_{23}$ represent the shear stress components. The material strength parameters include $X_{t}$ and $X_{c}$ for longitudinal tension and compression, $Y_{t}$ and $Y_{c}$ for transverse tension and compression. Additionally, $S_{12}$, $S_{13}$, and $S_{23}$ denote the shear strengths in the respective planes. Composite laminates may experience multiple concurrent failure modes. Damage initiation occurs when any of the failure criteria ($F_{ft}$, $F_{fc}$, $F_{mt}$, $F_{mc}$) attains a value of 1, triggering stiffness degradation in the affected element.

## 3. Finite Element Simulation Analysis

### 3.1. Finite Element Model Establishment and Material Parameters

To investigate the damage evolution behavior of carbon fiber reinforced polymer laminates under axial tensile loading in both intact and defected conditions, tensile failure simulations were conducted using the 3D Hashin failure criteria. Two three-dimensional solid models with dimensions of 150 mm × 20 mm × 3 mm were established in ABAQUS 2022. Both laminate models employed the same stacking sequence of [(90, 0)_5_, (0, 90)_5_], with a single ply thickness of 0.15 mm, resulting in a total of 20 plies.

**Figure 3:**
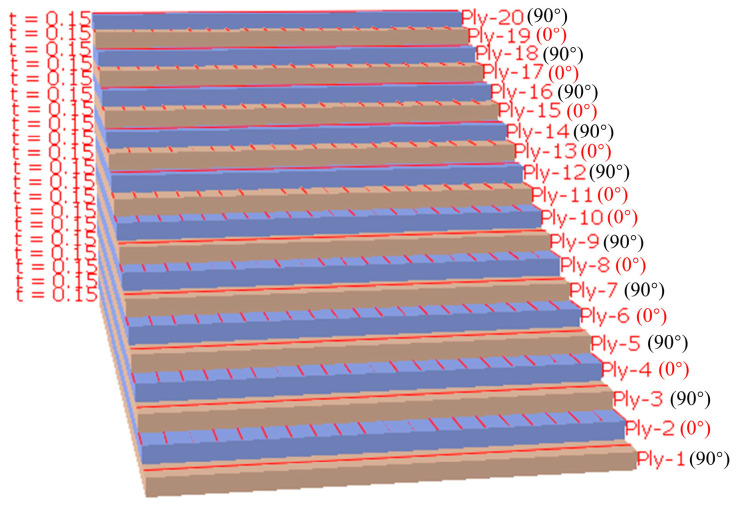
Angle of ply stacking between laminate layers.

**Figure 4:**
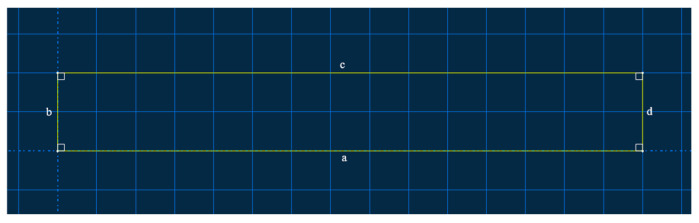
Intact model cross-sectional sketch.

**Figure 5:**
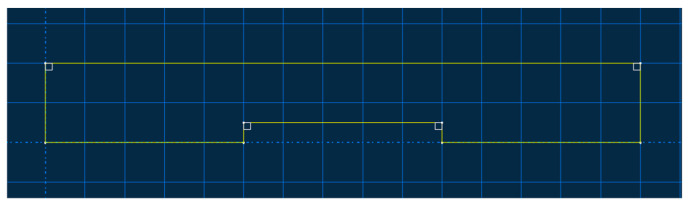
Defective model cross-sectional sketch.

**Figure 6:**
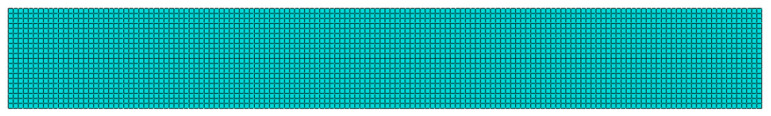
Intact model mesh diagram.

**Figure 7:**
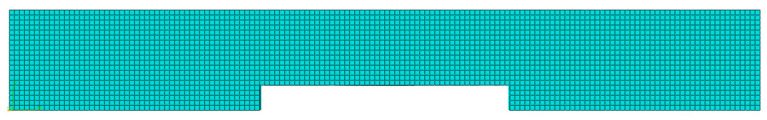
Defective model mesh diagram.

The two models were respectively configured as an intact specimen and a specimen with a prefabricated defect measuring 50 mm × 5 mm. These were designated as S1 (intact specimen) and S2 (specimen containing a 50 mm × 5 mm defect). The interlaminar stacking configuration of the laminates is shown in Figure 3. The modeling approach involves creating a 3D deformable solid extrusion model in ABAQUS. First, a 150 mm × 20 mm rectangular cross-section is sketched, as shown in Figure 4. The intact specimen model is obtained by extruding this sketch by 3 mm. Next, a 50 mm × 5 mm rectangular defect is introduced on side a of the cross-section sketch, while sides b, c, and d remained unchanged.. Unnecessary edges are removed using automatic trimming, as shown in Figure 5. Finally, the specimen model with a prefabricated defect is generated by extruding the modified sketch by 3 mm. Mesh generation for both the intact specimen and the notched specimen is depicted in the accompanying Figure 6 and Figure 7.

The material density, elastic properties, and 3D Hashin failure criteria parameters used in the simulation are listed in Table 1. In both CFRP laminate models, the left end was fully constrained, while a displacement load of 10 mm along the X-axis was applied to the right end.

### 3.2. Simulation Results and Analysis

**Figure 8:**
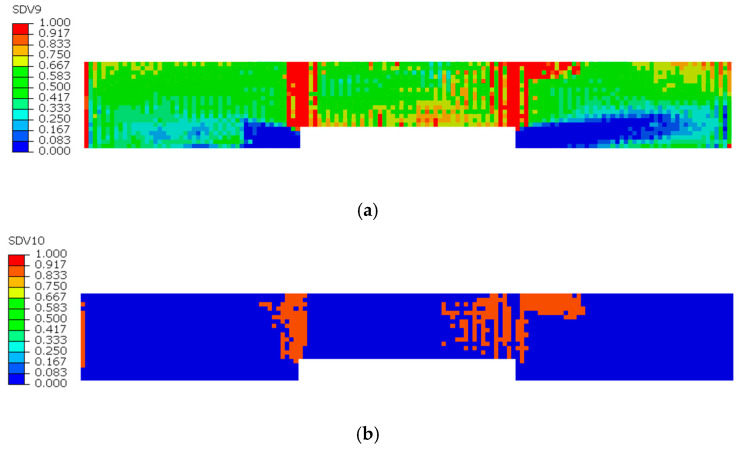
Tensile and compressive damage in the 90° sublayer matrix: (**a**) Tensile damage of the 90° sublayer matrix; (**b**) Compressive damage of the 90° sublayer matrix.

**Figure 9:**
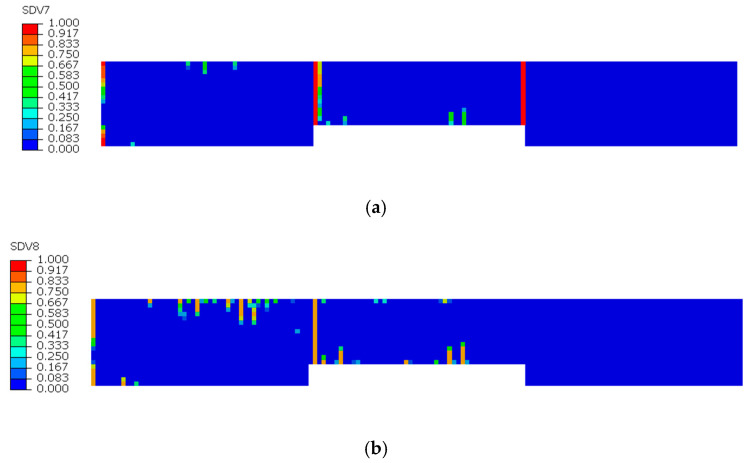
Tensile and compressive damage of 0° sublayer fibers: (**a**) Tensile damage of 0° sublayer fibers; (**b**) Compressive damage of 0° sublayer fibers.

**Figure 10:**
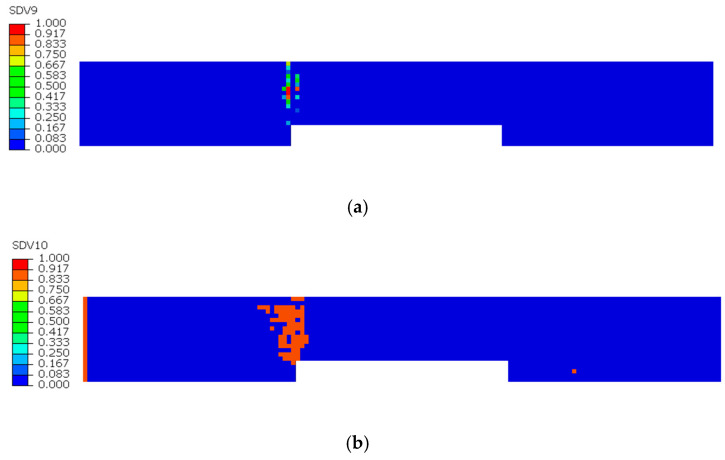
Tensile and compressive damage in the 0° sublayer matrix: (**a**) Tensile damage of the 0° sublayer matrix; (**b**) Compressive damage of the 0° sublayer matrix.

Figure 8, Figure 9 and Figure 10 illustrate the damage evolution of model S2 during the tensile process, where SDV7–SDV9 represent fiber tensile damage, fiber compressive damage, matrix tensile damage, and matrix compressive damage, respectively. The values in the legend indicate the damage state of the CFRP laminate, with 0 representing no damage and 1 indicating complete failure.

First, analyze the damage evolution in the 90° sublayer in Figure 8a,b. In this figure, since the fiber direction of the 90° sublayer is perpendicular to the applied tensile load, the fibers barely participate in bearing the load. Instead, the external load is primarily carried by the matrix. As a result, no fiber damage occurs in this layer, and the damage is mainly matrix-dominated. The presence of defects compromises the material’s integrity, leading to stress concentration near the notches during tension. Consequently, matrix tensile and compressive damage appear in the vicinity of the notches, with the most severe damage occurring at the notch edges.

In Figure 8a, concentrated red zones (where red indicates complete damage in the laminate) can be observed near the notch, indicating a higher degree of matrix tensile damage compared to other regions. In contrast, the orange zones near the notch in Figure 8b signify the presence of matrix compressive damage. However, the severity of this compressive damage is lower than that of the tensile damage, so the fracture in the 90° sublayer of the model is primarily induced by tensile damage.

The damage evolution in the 0° sublayer is analyzed based on Figure 9 and Figure 10. Figure 9a reveals that fiber tensile damage primarily concentrates at the defect edges, exhibiting complete damage (red) that fully penetrates the specimen. In contrast, Figure 9b shows fiber compressive damage is present but less severe than tensile damage, not reaching complete failure. Figure 10a,b demonstrates that both matrix tensile and compressive damage in the 0° sublayer are localized near the left defect edge, though their extent is notably less severe compared to fiber tensile damage. The analysis indicates that stress concentration induced by defects leads to concurrent initiation of four damage modes in the 0° sublayer: fiber tensile/compressive damage and matrix tensile/compressive damage. These damages predominantly originate from notch edges, with fiber tensile damage being the dominant failure mode. This damage mechanism fundamentally differs from that observed in defect-free specimens, where stress distribution and damage progression follow different patterns.

**Figure 11:**
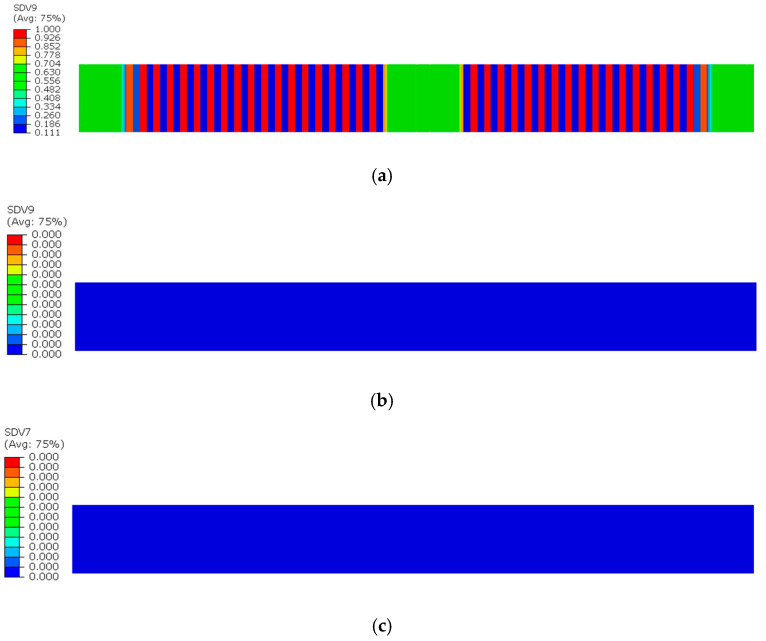
Damage state diagrams of each layer at the moment of complete matrix failure in the 90° sublayer of the intact specimen: (**a**) Matrix tensile damage in the 90° sublayer; (**b**) Matrix tensile damage in the 0° sublayer; (**c**) Fiber tensile damage in the 0° sublayer.

During the tensile damage process of intact specimens, matrix tensile damage first initiates and progressively expands within the 90° sublayer. Notably, neither matrix tensile damage nor fiber tensile damage occurs in the 0° sublayer until the 90° sublayer experiences complete matrix failure. As shown in Figure 11a, multiple locations in the 90° sublayer exhibit full matrix failure. Correspondingly, Figure 11b,c demonstrates that both matrix tensile damage and fiber tensile damage values remain zero across all positions in the 0° sublayer, indicated by a uniform dark blue coloration.

**Figure 12:**
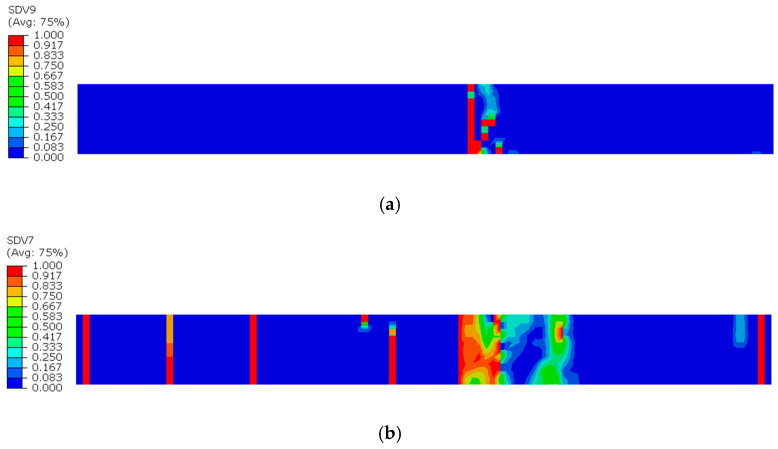
Complete failure diagram of the 0° sublayer in the intact specimen: (**a**) Matrix tensile damage in the 0° sublayer; (**b**) Fiber tensile damage in the 0° sublayer.

Subsequently, damage begins to develop in the 0° sublayer. Since the fiber direction in the 0° plies is parallel to the tensile load, the fibers primarily bear the applied load. As the tensile load continues to increase, the fiber stress gradually approaches its ultimate strength, eventually leading to fiber fracture in multiple regions, as shown in Figure 12. These fracture areas are randomly distributed, differing from the defective specimens, where fiber fractures are concentrated at the defect edges. When both fiber tensile damage and matrix tensile damage in the 0° plies reach a value of 1 and form continuous failure zones within localized areas, the ply is considered completely failed. As observed in Figure 12, through-thickness failure occurs at a position slightly to the right of the mid-region in the 0° ply.

In summary, there are fundamental differences in the damage evolution mechanisms between defective and intact CFRP laminates. For intact CFRP laminates, the failure process is characterized by the initial occurrence of matrix cracking in the 90° plies, which causes local stress concentration and subsequently promotes damage propagation to the adjacent plies. After complete failure of the 90° plies, damage begins to initiate in the 0° plies. In this case, the damage locations in the 0° plies are randomly distributed. When both the fiber tensile damage and matrix tensile damage reach a value of 1 and form through-thickness failure zones in localized areas, the 0° plies are considered fully failed, ultimately resulting in the complete failure of the laminate.

In contrast, the defective CFRP laminates exhibit a multi-mechanism cooperative damage propagation behavior due to stress concentration effects around the notch. Damage is predominantly concentrated at the notch edge, where fiber tensile damage is the primary failure mode, accompanied by matrix compressive damage, matrix tensile damage, fiber compressive damage, and other coupled damage phenomena. This forms a complex interactive damage mechanism around the defect region. Therefore, to effectively monitor the entire damage process in notched CFRP laminates, it is recommended that embedded FBG sensors be preferentially positioned near the notch edge in subsequent experiments, enabling the capture of critical damage evolution behaviors at key locations.

## 4. Experiment and Analysis

### 4.1. Preparation of Experimental Specimens

The laminates were fabricated using T700 carbon fiber unidirectional prepreg with dimensions of 150 × 20 mm. Each ply had a thickness of 0.15 mm, totaling 20 plies for an overall thickness of 3 mm. The stacking sequence is [(90, 0)_5_, (0, 90)_5_], which is consistent with the finite element simulation model.

When laying the unidirectional prepreg plies up to the 10th layer, the FBG is placed in the middle. The laying process then continues with the remaining plies. Since the optical fiber is relatively fragile, it is sleeved before placement to effectively protect it from breakage. The FBG sensor (Jinan Dahui Protoelectric Technology Co., Jinan, China) used has a grating length of 10 mm, a reflectivity greater than 90%, a side-mode suppression ratio of more than 15 dB, an acrylate coating, and the fiber type is SMF-28E. After completing the layup, the laminate is cured using a hot press at a curing temperature of 130 °C.

**Figure 13:**
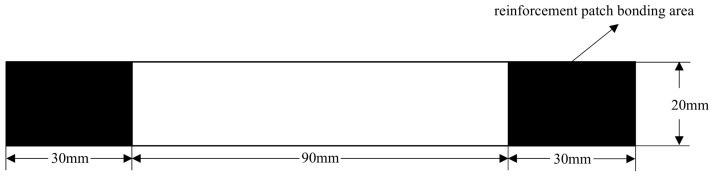
Schematic diagram of the first complete set of specimens.

**Figure 14:**
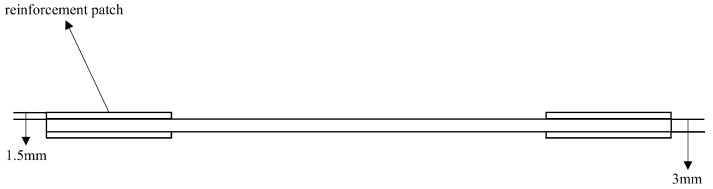
Schematic diagram of the thickness direction of the first complete set of specimens.

**Figure 15:**
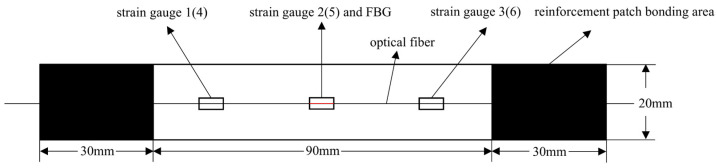
Schematic diagram of the second group of complete specimens and sensor layout.

**Figure 16:**
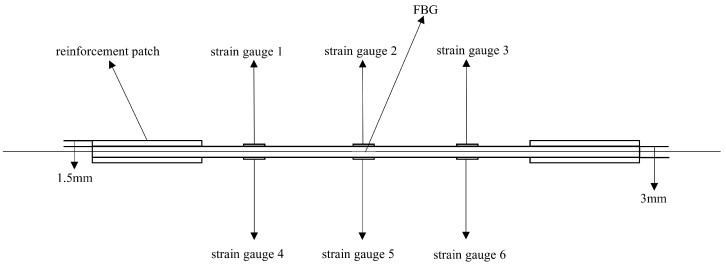
Schematic diagram of the thickness direction of the second group of complete specimens.

**Figure 17:**
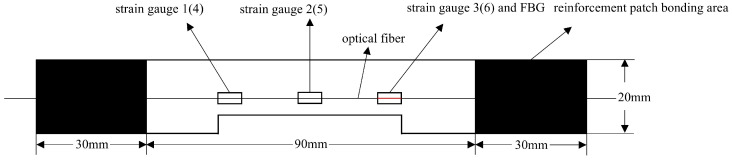
Schematic diagram of the third group of defective specimens and sensor layout.

**Figure 18:**
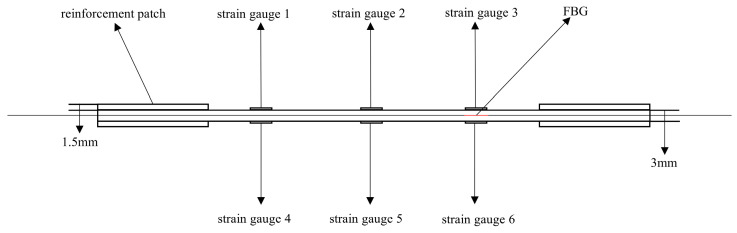
Schematic diagram of the thickness direction of the third group of defective specimens.

Three sets of specimens were prepared as shown in Figure 13, Figure 14, Figure 15, Figure 16, Figure 17 and Figure 18. The first and second sets consisted of undamaged specimens, while the third set contained specimens with a 50 × 5 mm artificial defect introduced by post-cure machining. To prevent damage to the gripping ends during subsequent tensile testing on a hydraulic machine, reinforcing tabs were bonded to the specimen clamping regions using LOCTITE E-20HP epoxy adhesive.

The first set comprised three specimens without any surface-mounted strain gauges or embedded FBG sensors. The second set included three specimens instrumented with BE120-3AA-P2K(GUOXINGHUAYU, Beijing, China) resistance strain gauges on the surface and an FBG sensor embedded between the 10th (0° ply) and 11th (0° ply) layers at the center. The third set contained two defective specimens, similarly equipped with surface-mounted BE120-3AA-P2K strain gauges and an embedded FBG sensor between the 10th and 11th layers. The specimen configuration and sensor arrangement are illustrated in the accompanying figures, with detailed specifications provided in Table 2.

### 4.2. Test System Description

In this study, tensile failure experiments were conducted on CFRP laminates with dimensions of 150 mm × 20 mm × 3 mm. During the tensile tests, strain data from both surface-mounted electrical resistance strain gauges and embedded FBG sensors were collected. The strain gauge data were acquired using a GH3840 dynamic and static signal testing system(GUOXINGHUAYU, Beijing, China), while the FBG strain data were recorded by a custom-built fiber Bragg grating demodulation device. All acquired data were processed and archived using corresponding computer software.

At room temperature, a tensile test was conducted using an Instron 8801 servo-hydraulic testing machine(INSTRON, Shanghai, China). The machine’s grips clamped the reinforced sections at both ends of the specimen to secure it. The loading rate was set to 0.5 mm/min. During the tensile process, the data from surface-mounted strain gauges, embedded FBG sensors, and the testing machine’s load were monitored in real-time through demodulation software on the computer and saved. Each saved data point includes the operating system time to facilitate subsequent data synchronization.

**Figure 19:**
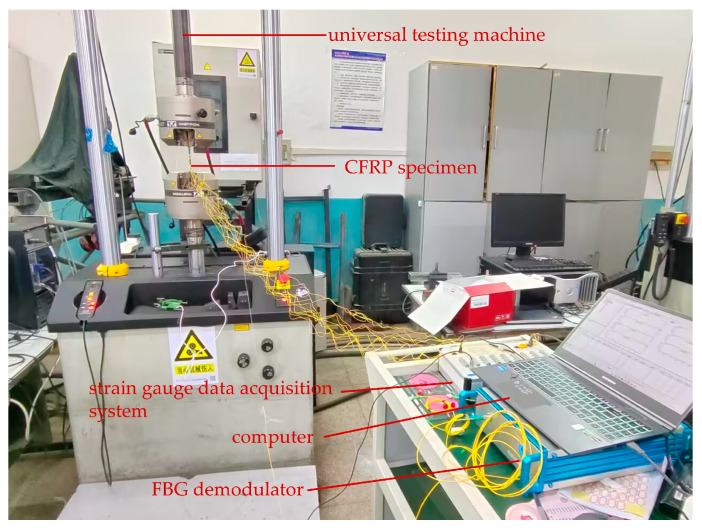
CFRP laminate mechanical testing system.

The overall composition of the testing system is shown in Figure 19, which mainly includes the tensile loading equipment and the data acquisition units.

### 4.3. Compatibility Testing of FBG with Composite Laminates

Currently, the most commonly used FBG sensors have diameters ranging from 125 to 250 μm. Compared to reinforcement fibers with diameters as small as 10 μm, this relatively large size may cause damage to the internal structure of composite materials when embedded, leading to localized stress concentrations and potentially adverse effects on the overall mechanical properties of the structure.

To investigate whether embedded FBG sensors affect CFRP laminates, we prepared two groups of test specimens with identical dimensions: one group without embedded FBG sensors and the other with embedded FBG sensors. Tensile failure tests were then conducted on both groups. The experimental results are presented in Table 3.

Table 3 presents a comparison of failure loads between specimens with embedded FBG sensors and those without. The average failure load for non-embedded specimens was 20,741 N, while for FBG-embedded specimens it was 19,884 N, representing a 4% reduction in load-bearing capacity. This difference indicates that although FBG sensor embedding does affect the structural integrity of laminates to some extent, its weakening effect on mechanical properties is relatively minor. Consequently, the impact of FBG sensor embedding on the tensile performance of CFRP laminates can be considered negligible in practical applications.

### 4.4. Test Data Analysis of Intact Specimens

**Figure 20:**
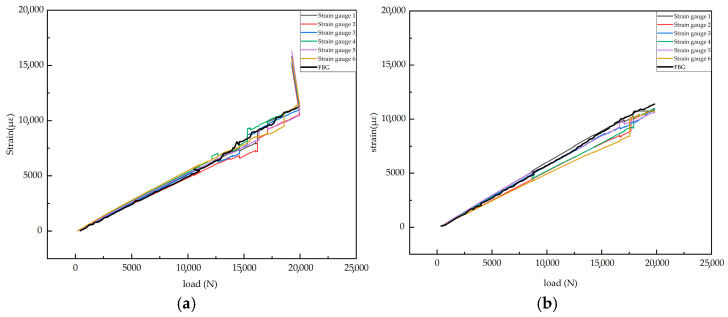
Strain versus load curves: (**a**) Specimen 2-1; (**b**) Specimen 2-2.

A detailed analysis was conducted using the tensile test data of specimen 2-1. Figure 20a presents the strain variation measured by the surface-mounted resistance strain gauge and the strain evolution recorded by the embedded FBG sensor under increasing load. To ensure reproducibility, the data graph for specimen 2-2 is also provided in Figure 20b. It can be observed that the trend is similar to that of specimen 2-1, indicating good repeatability.

**Figure 21:**
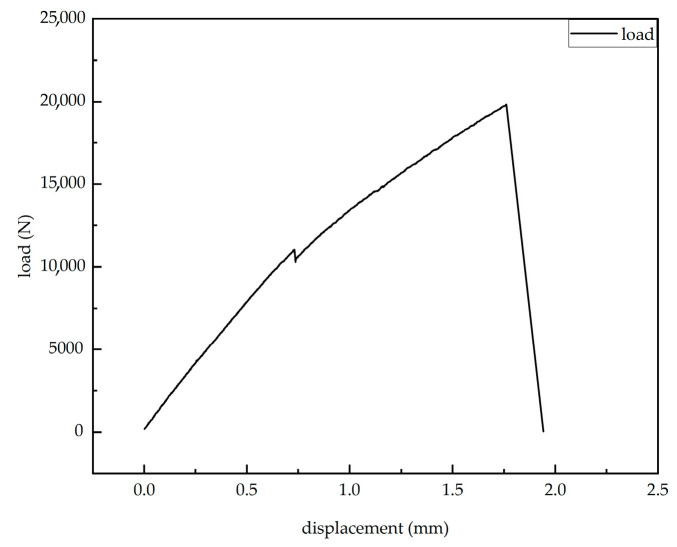
Tensile testing machine load–displacement curve.

The strain–load relationship diagram measured by the resistance strain gauges shows that when the load is less than 10,757 N, the strain on the surface of the laminate exhibits a linear relationship with the load. The slopes from all strain gauges are similar, with the minimum slope around 0.50 and the maximum slope approximately 0.54. This indicates that the laminate surface is in the elastic deformation stage when the load is below 10,757 N. When the load reaches 10,757 N, a phenomenon occurs where the load remains constant or even decreases while the strain changes dramatically, showing nonlinear behavior. According to the load–displacement curve provided by the tensile machine (Figure 21), the curve maintains a linear increase before 10,757 N. At 10,757 N, the displacement suddenly increases while the load drops. Both the tensile machine’s load–displacement curve and the strain–load curve from the resistance strain gauges exhibit abrupt jumps near this load, ruling out the possibility of fixture slippage. This indicates that when the load reaches 10,757 N, damage initiates in the near-surface region of the composite laminate, leading to a reduction in the material’s load-bearing capacity and a sudden local stiffness drop, resulting in strain concentration.

In the range of 10,757 N to 19,983 N, the strain–load curves of all strain gauges show multiple step-like jumps. During these jumps, the load changes minimally while the strain surges abruptly. This is a typical characteristic of composite material damage, involving phenomena such as matrix cracking and localized fiber breakage, which cause load release in the damaged area and stress redistribution, manifesting as sudden jumps in strain values. Finally, at the ultimate load, the strain increases sharply while the load slightly decreases. This indicates that in the final failure stage, the laminate does not fracture instantaneously but rather partially fractures first, with the remaining sections continuing to bear the load before eventually failing. Thus, the overall stiffness gradually decreases, the load drops, and the deformation (strain) grows rapidly.

The strain–load relationship diagram measured by the FBG shows that when the load is less than 11,030 N, the strain–load relationship is linear, indicating that the central region of the laminate is in the elastic deformation stage without internal damage. When the load reaches 11,030 N, a phenomenon occurs where the load remains constant or decreases while the strain changes dramatically, suggesting that microscopic damage begins to develop at the interlaminar region where the FBG is located. The subsequent curve exhibits clear nonlinearity, with small abrupt jumps and sudden increases, indicating that the interlaminar damage detected by the FBG sensor is continuously expanding. Finally, the central wavelength of the FBG can no longer be captured after the load exceeds 19,717 N, which is likely due to fiber breakage in the internal layers of the laminate and damage to the FBG grating region, rendering it unable to continue collecting signals.

A comparison between the resistance strain gauge and FBG data reveals that when an intact composite laminate is subjected to tensile loading, the failure mode begins with matrix damage on the surface, leading to localized stress concentrations. Subsequently, internal matrix damage occurs within the laminate, and finally, with the continued accumulation of damage, the laminate undergoes complete failure. This failure progression aligns closely with the simulation results.

### 4.5. Analysis of Test Data for Specimens with 50 × 5 mm Defects

**Figure 22:**
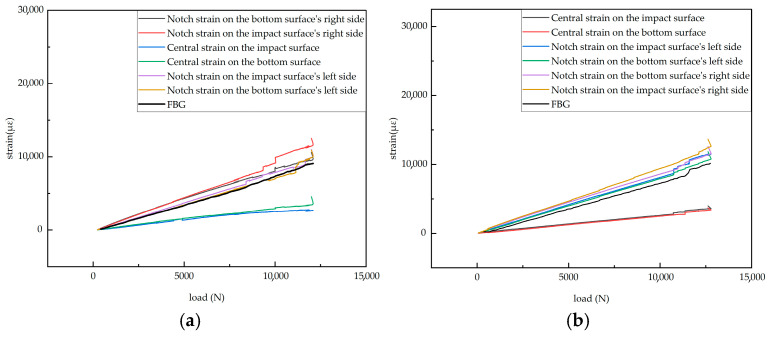
Strain versus load curves for defective specimens: (**a**) Specimen 3-1; (**b**) Specimen 3-2.

To ensure repeatability, this study conducted tensile tests on two 50 × 5 mm defective specimens. The experimental data plots for both tests are provided, showing similar trends between the two experiments, which indicates good repeatability. A detailed analysis was performed on the data in Figure 22a. Figure 22a shows the load-dependent strain variation measured by surface-mounted resistance strain gauges and the corresponding strain evolution recorded by embedded FBG sensors during the tensile test of a specimen with a 50 × 5 mm defect.

In Figure 22a, the specific arrangement of the six strain gauges is shown in Figure 17 and Figure 18, exhibiting a pairwise symmetrical distribution. The strain–load curves reveal that before the load reaches 8000 N, the strain at all measurement points changes linearly with the load, indicating that despite structural defects such as notches in the laminated plate, the material primarily remains in an elastic deformation state during the low-load stage. Further comparison of the strain–load response coefficients at each measurement point shows that the strain values on the left and right sides are greater than those at the central positions, and the response coefficients of symmetrically arranged strain gauges on the surface and bottom layers are essentially similar. This reflects the non-uniform stress distribution characteristics at different positions on the material surface. Notably, due to the significant stress concentration effect at the edges of the notches, the strain–load response coefficients in these areas are markedly higher than those in the central regions away from the notches.

When the load exceeds 8000 N, the strain–load curves at the left and right measurement points successively exhibit multiple step-like abrupt changes—i.e., the strain values suddenly increase with little change in the load. This phenomenon indicates the initiation and propagation of cracks at the edges of the notches. Among these, the strain jumps at the two right measurement points are particularly pronounced, suggesting that cracks preferentially initiate on the right side of the notch. In contrast, the strain at the central measurement points maintains a linear relationship with the load, indicating that the structure in regions away from the notch remains intact and undamaged.

At the final stage of loading, when the applied load reached its ultimate value of 12,080 N, the strain values at all measurement points increased sharply, accompanied by a slight drop in load, indicating the overall failure of the specimen. This failure was associated with through-thickness cracking, interlaminar delamination of carbon fiber layers, matrix fracture, and structural instability.

**Figure 23:**
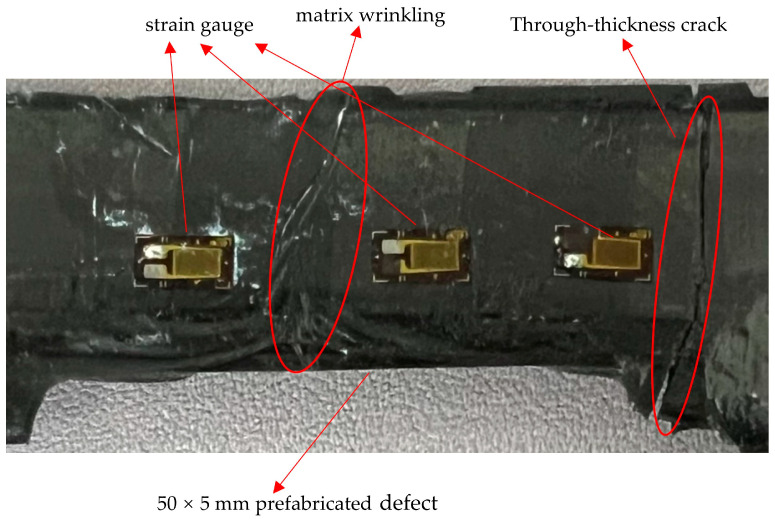
Fracture diagram of the specimen after tensile testing.

As shown in Figure 23, the fracture locations after tensile failure were primarily concentrated on the right side of the notch, while no through-thickness cracks were observed on the surface in the measurement area on the left side. Combined with the load–strain curve characteristics presented in Figure 22a, it can be concluded that cracks initially originated on the right side of the notch and propagated along the thickness direction, resulting in a local redistribution of the stress field. This redistribution caused a rapid stress increase in the left-side region near the measurement points. Although no through-thickness cracks formed on the left side, localized interlaminar delamination and fiber–matrix debonding were observed in this area.

Moreover, as shown in Figure 23, noticeable surface anomalies such as matrix wrinkling and resin uplift occurred near the left-side measurement points, with localized bulging or indentation, indicating the accumulation of internal damage. This damage accumulation triggered abrupt jumps in the strain response curves recorded at the left-side measurement points. In contrast, the central measurement points located outside the crack propagation path experienced relatively lower loading effects, with their strain responses maintaining good linearity throughout the loading process. This further confirmed that crack propagation predominantly extended along the right side of the notch without spreading to other areas of the laminate.

**Figure 24:**
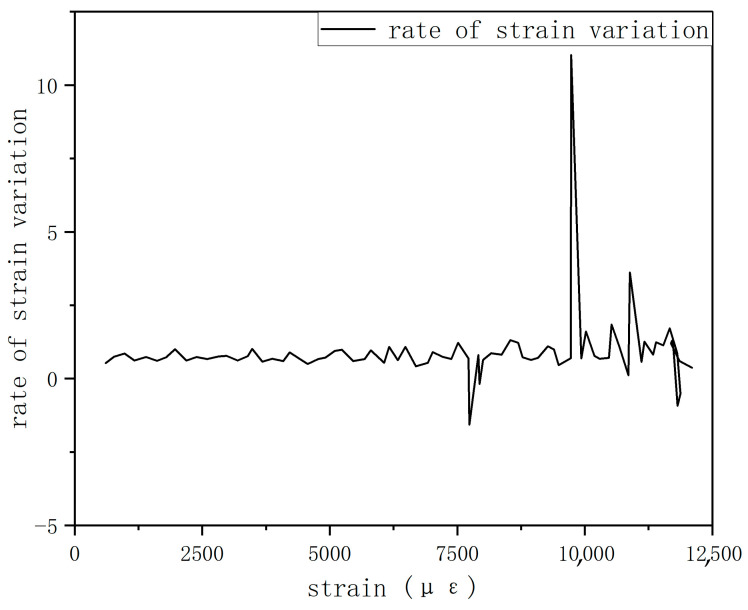
Strain rate variation diagram of FBG sensor.

In Figure 24, the black line represents the strain–load curve monitored by the FBG sensor at the right-side damage location embedded in the center layer of the composite laminate. To highlight minor strain variations, the strain rate curve of this strain–load curve was calculated to amplify the subtle changes. As shown in Figure 24, a noticeable change in the strain rate is observed near a load of approximately 8000 N: before this point, the strain rate remains consistently positive, averaging about 0.8 με/N, but at 8000 N, it turns negative for the first time, reaching about −1.57 με/N. This suggests that initial damage, such as resin cracking, fiber/matrix interface debonding, or microcracks, may begin to occur in the local area. Although such damage causes only minor changes in the macroscopic strain curve, it significantly affects local stiffness, manifested as a sudden increase in the absolute value of the strain rate. The appearance of negative values indicates local relaxation or microstructural abrupt changes, meaning that despite the increasing load, the overall strain decreases instead.

A comparison between the FBG sensor monitoring results and the strain gauge data from the corresponding surface location reveals that both the surface and center-layer strain gauges exhibit abrupt strain jumps near 8 kN. Subsequently, strain jumps occur in the bottom-layer strain gauges after the load reaches 9.7 kN. Finally, when the load reaches 12,080 N, the FBG sensor’s central wavelength signal is interrupted, and data can no longer be collected. This is presumed to be due to the fracture of the specimen, causing the embedded optical fiber at the fracture location to break. This demonstrates that in defective laminates, both the internal layers and the surface layers can serve as initiation sites for damage.

### 4.6. Defect Monitoring Analysis of CFRP Laminates

Based on the analysis results presented in Section 4.4 and Section 4.5, it can be concluded that both defective and intact CFRP laminates experienced a distinct elastic deformation stage prior to the initiation of the first crack. To further investigate the specific influence of defects on the mechanical response characteristics of CFRP laminates during the elastic stage, this study extracted the strain–load data recorded by embedded FBG sensors in both intact and defective laminates within the elastic range. Linear fitting was then performed on the strain–load data for both cases.

**Figure 25:**
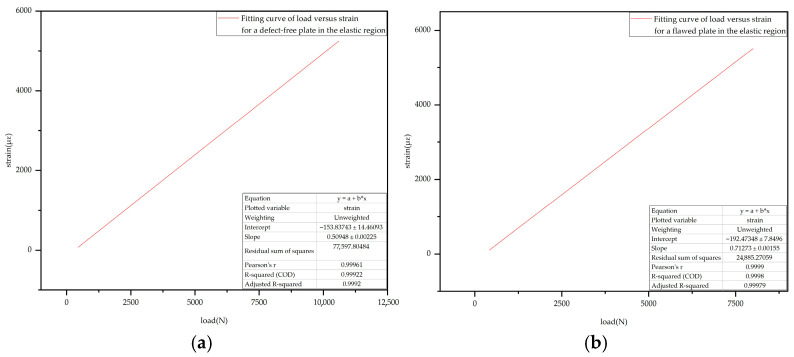
Strain–load curve during the elastic stage: (**a**) Intact laminate; (**b**) Defective laminate.

The fitting results are presented in Figure 25, where Figure 25a shows the linear fitting result for the strain–load curve of the intact laminate during the elastic stage, while Figure 25b displays the corresponding result for the defective laminate under the same conditions.

From the fitting results, it can be observed that the slope of the strain–load curve for the intact laminate during the elastic stage is approximately 0.51, while that of the defective laminate is about 0.71, indicating a significant difference between the two. This result suggests that the presence of a defect notably alters the load–strain response characteristics of the laminate in the elastic stage. Specifically, the defect weakens the local structural stiffness, leading to stress concentration in the defective region under external loading. This, in turn, reduces the local deformation capacity, accelerates the overall strain response rate, and ultimately results in an increased slope in the fitted curve. This phenomenon not only reveals the mechanism by which defects affect the mechanical behavior of CFRP laminates during the elastic stage but also provides an important basis for defect identification and monitoring in practical engineering applications.

Based on this aforementioned characteristic, the high sensitivity and real-time monitoring capability of embedded FBG sensors can be fully exploited to achieve continuous online monitoring of the strain response in CFRP laminates during service. When an abnormal increase in the slope of the strain–load curve is detected during the elastic stage, it indicates the presence of internal defects such as localized material loss, delamination, or microcracks within the structure, suggesting a reduction in structural stiffness. This real-time strain monitoring approach based on embedded FBG sensors provides dynamic structural health information for critical load-bearing components of aerospace vehicles. Consequently, it enables timely adjustment of flight attitude and optimization of load distribution strategies under complex service conditions, preventing catastrophic structural failure caused by the progressive accumulation of localized damage, thereby effectively ensuring the structural safety and service reliability of the aerospace vehicle.

## 5. Conclusions

In this study, the progressive damage mechanisms of defective CFRP laminates under tensile loading were systematically investigated through finite element simulations combined with experimental tests using surface-mounted electrical resistance strain gauges and embedded FBG sensors. A comparative analysis with intact laminates was conducted to validate the effectiveness of the proposed multi-sensor monitoring approach for tracking damage evolution in defective laminates. The main conclusions are as follows:

(1) For laminated plates containing defects, surface-mounted resistance strain gauges and embedded FBG sensors can effectively monitor the evolution of strain around the defective area. Experimental results indicate that significant stress concentration is prone to occur at the edge of the defect, making it the initial damage zone. Both the surface strain gauges and FBG sensors detected a sudden strain jump at a load of 8 kN, while the underlying strain gauge began to register the strain jump at 9.7 kN. This demonstrates that the presence of defects randomizes the initiation layer of damage, but the damage location is consistently at the edge of the defect. Ultimately, under critical loading, the laminated plate fractured at the defect edge, leading to the interruption of monitoring signals. The observed damage pattern aligns well with the simulation results.
(2) A comparison between the data of laminates with defects and intact laminates reveals that intact laminates typically first experience matrix cracking in the near-surface regions, leading to local stress concentration. Subsequently, damage occurs in the inner layers, meaning surface layer damage precedes inner layer damage. Ultimately, the accumulation of damage results in the overall failure of the laminate, with the location of through-thickness cracks exhibiting a certain degree of randomness. In contrast, the presence of defects disrupts this randomness, causing the fracture locations in defective laminates to often concentrate near the defect edges, and the damage zones tend to become more deterministic.
(3) The multi-sensor cooperative monitoring proposed in this paper can effectively meet the demand for dynamic monitoring of damage states in load-bearing aerospace structures. Embedded FBG sensors can monitor whether defects exist in the laminate in real time and sensitively track internal strain changes near the defect area as well as crack propagation in defective laminates, enabling real-time monitoring and early warning of internal damage evolution. Meanwhile, surface-mounted resistance strain gauges can promptly capture early-stage damage signals on the surface. The combined deployment of these two sensor types allows for multi-dimensional and multi-layer damage monitoring of defective laminates, effectively enhancing the reliability and coverage of structural health monitoring systems. Moreover, this approach offers advantages such as fast response, high monitoring accuracy, and minimal interference with the host structure, making it particularly suitable for real-time monitoring and early warning of complex damage states in high-performance composite structures. Its application not only enriches the technical methods for health monitoring of carbon fiber composite structures but also provides essential technical support and practical value for the safe service management of complex equipment, presenting broad engineering application prospects and potential for widespread adoption.

## Figures and Tables

**Table 1:** Material parameters of carbon fiber composite laminates.

Parameter	Value
ρ (kg/m^3^)	1600
E_1_/GPa	130
E_2_, E_3_/GPa	7.1
G_12_, G_13_/GPa	3.6
G_23_/GPa	3.08
μ_12_, μ_13_	0.32
μ_23_	0.52
X_T_/MPa	1760
X_C_/MPa	1100
Y_T_, Z_T_/MPa	51
Y_C_/Z_C_/MPa	167
S_12_, S_13_, S_23_/MPa	70

**Table 2:** Introduction of test pieces.

Test Group	Test Specimen Number	Dimension (mm)	Defect Condition
Group 1	S1-1, S1-2, S1-3	150 × 20 × 3	no defects
Group 2	S2-1, S2-2, S2-3	150 × 20 × 3	no defects
Group 3	S3-1, S3-2	150 × 20 × 3	50 × 5 mm defects

**Table 3:** Comparison of failure loads between FBG-embedded and non-embedded specimens.

Test Group	Sample Type	Failure Load	Average Failure Load
S1-1	no embedded FBG	20,619 N	20,714 N
S1-2	no embedded FBG	20,731 N	
S1-3	no embedded FBG	20,792 N	
S2-1	embedded FBG	19,983 N	19,884 N
S2-2	embedded FBG	19,813 N	
S2-3	embedded FBG	19,857 N	

## Data Availability

Restrictions apply to the datasets. The dataset presented in this article is not readily available as the data are part of an ongoing follow-up study. For requests to access the dataset, please contact yanguang79@bistu.edu.cn.
